# Burden of community diarrhoea in developing countries

**DOI:** 10.1016/S2214-109X(15)00247-8

**Published:** 2015-12-21

**Authors:** Tess Baker

**Affiliations:** a Peninsula Health Physiotherapy, Wantirna, Melbourne, VIC 3144, Australia

The Article by James Platts-Mills and colleagues (September, 2015),^1^ highlights the burden of diarrhoeal disease in young children in developing countries and also demonstrates the effect that seasonal variations have on multiple causative pathogens. Infectious gastroenteritis contributes significantly to the 1 billion episodes of diarrhoea and 3 million deaths in children under 5 years, and is the fifth leading cause of death worldwide.^2^

Pathogens causing diarrhoea frequently show seasonality, suggesting that climate and enteric disease are inextricably linked. This link has important implications if we accept the very real threat of climate change on human health. WHO quantified the impact of global warming on diarrhoea, reporting that warming by 1°C was associated with a 5% increase in diarrhoea.^3^ Increased rainfall has been associated with higher incidence of norovirus^4^ whereas rotavirus often peaks in colder months. As such, extreme weather events associated with climate change are likely to alter patterns of gastroenteritis. The increased replication rate of some bacterial and viral pathogens in warm conditions,^5^ combined with poor water and sanitation infrastructure, means that people in developing nations are particularly vulnerable.

Predicting the potential effects of climate change on the incidence and distribution of infectious gastroenteritis can assist public health providers to control and prevent severe outbreaks in the future. Implementation of programmes for rotavirus vaccination clearly shows benefit and should be an adaptation strategy to help cope with climate change. More importantly, however, Platts-Mills and colleagues^1^ identified multiple pathogens contributing to diarrhoeal disease, highlighting the necessity for a broader mitigation plan.
